# Effects of Allicin on Pathophysiological Mechanisms during the Progression of Nephropathy Associated to Diabetes

**DOI:** 10.3390/antiox9111134

**Published:** 2020-11-15

**Authors:** Abraham Said Arellano-Buendía, Luis Gerardo Castañeda-Lara, María L. Loredo-Mendoza, Fernando E. García-Arroyo, Pedro Rojas-Morales, Raúl Argüello-García, Juan G. Juárez-Rojas, Edilia Tapia, José Pedraza-Chaverri, Laura Gabriela Sánchez-Lozada, Horacio Osorio-Alonso

**Affiliations:** 1 Department of Cardio-Renal Physiopathology, Instituto Nacional de Cardiología “Ignacio Chávez”, México City 14080, Mexico; abraham.arellano@cardiologia.org.mx (A.S.A.-B.); lugecalas@gmail.com (L.G.C.-L.); enrique.garcia@cardiologia.org.mx (F.E.G.-A.); pedrorojasm@outlook.com (P.R.-M.); edilia.tapia@cardiologia.org.mx (E.T.); laura.sanchez@cardiologia.org.mx (L.G.S.-L.); 2 Histopathology Laboratory, Research Subdivision, School of Medicine, Universidad Panamericana, Donatello 43, Mexico City 03910, Mexico; lloredo@up.edu.mx; 3 Departamento de Biología, Facultad de Química, Universidad Nacional Autónoma de México, Ciudad de México 04510, Mexico; pedraza@unam.mx; 4 Departamento de Genética y Biología Molecular, Centro de Investigación y de Estudios Avanzados del Instituto Politécnico Nacional, Mexico City 07360, Mexico; rag@cinvestav.mx; 5 Department of Endocrinology, Instituto Nacional de Cardiología “Ignacio Chávez” México City 14080, Mexico; gabriel.juarez@cardiologia.org.mx

**Keywords:** diabetes, allicin, nephropathy, oxidative stress, fibrosis, hypoxia

## Abstract

This study aimed to assess the impact of allicin on the course of diabetic nephropathy. Study groups included control, diabetes, and diabetes-treated rats. Allicin treatment (16 mg/kg day/p.o.) started after 1 month of diabetes onset and was administered for 30 days. In the diabetes group, the systolic blood pressure (SBP) increased, also, the oxidative stress and hypoxia in the kidney cortex were evidenced by alterations in the total antioxidant capacity as well as the expression of nuclear factor (erythroid-derived 2)-like 2/Kelch ECH associating protein 1 (Nrf2/Keap1), hypoxia-inducible factor 1-alpha (HIF-1α), vascular endothelial growth factor (VEGF), erythropoietin (Epo) and its receptor (Epo-R). Moreover, diabetes increased nephrin, and kidney injury molecule-1 (KIM-1) expression that correlated with mesangial matrix, the fibrosis index and with the expression of connective tissue growth factor (CTGF), transforming growth factor-β1 (TGF-β1), and α-smooth muscle actin (α-SMA). The insulin levels and glucose transporter protein type-4 (GLUT4) expression were decreased; otherwise, insulin receptor substrates 1 and 2 (IRS-1 and IRS-2) expression was increased. Allicin increased Nrf2 expression and decreased SBP, Keap1, HIF-1α, and VEGF expression. Concurrently, nephrin, KIM-1, the mesangial matrix, fibrosis index, and the fibrotic proteins were decreased. Additionally, allicin decreased hyperglycemia, improved insulin levels, and prevented changes in (GLUT4) and IRSs expression induced by diabetes. In conclusion, our results demonstrate that allicin has the potential to help in the treatment of diabetic nephropathy. The cellular mechanisms underlying its effects mainly rely on the regulation of antioxidant, antifibrotic, and antidiabetic mechanisms, which can contribute towards delay in the progression of renal disease.

## 1. Introduction

Diabetes is a metabolic, chronic, progressive, and irreversible disease, characterized mainly by hyperglycemia. In addition, diabetes is one of the most important causes of cardiovascular diseases (CVD) and kidney complications, such as chronic kidney disease (CKD) and end-stage renal disease (ESRD) [1]. The proportion of ESRD attributable to diabetes alone ranges from 12 to 55% and the incidence of ESRD is up to 10 times higher in adults with diabetes as compared to non-diabetic adults [2]. In diabetic patients, CKD may be caused by diabetic nephropathy (DN), non-diabetic renal disease (NDRD), or a combination of both [3].

On the other hand diabetes, hypertension, dyslipidemia and smoking are key risk factors for CVD [4,5,6]. In fact, people with both diabetes and hypertension have a four-fold increased risk for the development of CVD and substantially higher risk of CVD death and all-cause mortality [4,7,8].

In diabetic patients normalizing blood sugar, the control of dyslipidemia, and reduction of blood pressure are the primary targets to improve life quality, to delay the progression to CKD, and prevent cardiovascular complications [1,2,3,5]. However, these actions do not prevent the progression to ESRD. Thus, several etiologies and mechanisms of pathogenesis and progression of DN have been postulated. Evidence from experimental and clinical studies of diabetes have shown that oxidative stress (OS) is associated with the initiation and progression of vascular complications, including diabetic nephropathy and CVD [9,10,11,12,13,14].

Furthermore, glucose metabolism and increased tubular reabsorption due to hyperfiltration during diabetes can increase oxygen consumption in the proximal tubule resulting in renal hypoxia [15]. Other studies report that renal hypoxia is an early manifestation of CKD progression and a common pathway for nephropathy, particularly tubulointerstitial hypoxia, which is closely related to OS and the development of diabetic complications [16,17,18].

The kidney exhibits high sensitivity to changes in oxygen pressure, making it highly susceptible to hypoxia. Further, it has been hypothesized that OS modulates hypoxic factors. Hypoxia inducible factor-1α (HIF-1α) is the main regulator of cellular response to hypoxia modulating multiple cellular processes in the kidney [15,19]. Under hypoxic conditions, HIF-1α modulates apoptosis, autophagy, inflammation, and cell cycle arrest, among other cellular mechanisms that are involved in renal protection [20]. However, HIF-1α also contributes to interstitial fibrosis through the induction of profibrotic pathways, and therefore to the progression of kidney injury [20].

Current treatments aimed to prevent the progression of CKD might delay the diabetic nephropathy and other vascular complications; nevertheless, the disease burden remains high; thus, many patients with DN continue to progress to CKD, ESRD, or CVD [2,3]. Therefore, new approaches are needed to control OS overproduction, hypoxia, fibrosis, and pro-inflammatory cytokine production. In this regard, the use of plants or substances of natural origin with medicinal properties or therapeutic potential is under current study. Many natural compounds have antioxidant properties and display other biological activities in chronic diseases such as hypertension, diabetes, and metabolic syndrome [13].

Allicin is a natural compound produced from the stable precursor S-allyl cysteine-sulfoxide (alliin) by the action of the enzyme alliinase. This reaction occurs when garlic cloves are crushed or macerated [21]. This compound has shown various beneficial effects on chronic diseases. The biological activities from allicin include antimicrobial [22], antihypertensive [23,24], antioxidant [23,25], antidiabetic [25,26,27], cardioprotective [22,23,24,25], nephroprotective [23,24,26] as well as immunomodulator [22,27]. Several cellular mechanisms have been proposed to explain how allicin exerts its beneficial effects, but in renal fibrosis these are still unknown or poorly studied.

Thus, the present study aimed to assess if the natural antioxidant allicin may delay the progression of experimental diabetic nephropathy, as well as the possible mechanisms involved.

## 2. Materials and Methods

### 2.1. Reagents

2,2′-Azino-bis(3-ethylbenzothiazoline-6-sulfonic acid) diammonium salt (ABTS), dichloromethane, diallyl disulfide, hydrogen peroxide and streptozotocin (STZ), sodium dodecyl sulfate, sodium chloride, trolox, and cOmplete™ Protease Inhibitor Cocktail were purchased from Sigma-Aldrich (St. Louis, MO, USA). Antibodies to beta-Actin (GTX109639), connective tissue growth factor (CTGF) (GTX124232), glucose transporter 4 (GLUT4) (GTX79317), hypoxia-inducible factor 1-alpha (HIF-1α) (GTX127309), kidney injury molecule-1 (KIM-1) (GTX85067), kelch-like ECH associated-protein 1 (KEAP1) (GTX60660), erythropoietin (Epo) (GTX41256), erythropoietin receptor (Epo-R) (GTX37704), nuclear factor erythroid 2-related factor 2 (Nrf2) (GTX103322) and vascular endothelial growth factor (VEGF) (GTX21316) were acquired from GeneTex (Irvine, CA, USA) and those for nephrin (sc-377246), insulin receptor substrate-1(IRS-1) (sc-515017), insulin receptor substrate-2 (IRS-2) (sc-390761), and transforming growth factor-beta 1 (TGF-β1) (sc-130348) from Santa Cruz Biotechnology, Inc. (Dallas, TX, USA). Antibody to alpha-smooth muscle actin (α-SMA) (ab265588) was purchased from ABCAM (Cambridge, MA, USA.) Goat anti-rabbit and goat anti-mouse IgG-HRP were purchased from Cell Signaling Technology (Danvers, MA, USA). All other chemicals used were of the highest purity available. Commercial kits to measure blood urea nitrogen (BUN) and creatinine levels in plasma were obtained from SpinReact (Girona, Spain).

### 2.2. Experimental Design

**Figure 1:**
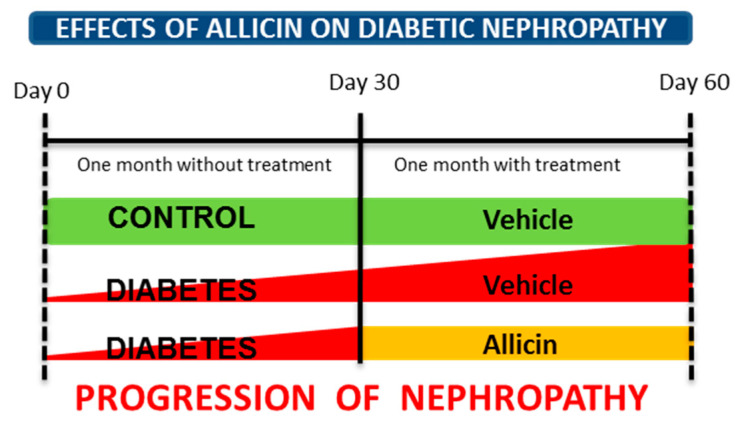
Experimental design to assess the effects of allicin on progression of diabetic nephropathy. After one month of follow-up, the treatments were started and given for one month.

Male Wistar rats weighing 280–310 g were used. Animals were randomly divided into three groups: control (Ctrl, *n* = 6), diabetes (DM, *n* = 7), and diabetes treated with allicin (DA *n* = 7). Diabetes was induced by a single administration of STZ (50 mg/kg i.p.) dissolved in citrate buffer (0.1 M, pH 4.5). We previously demonstrated histopathological alterations at glomerular and tubular levels in this experimental model [12]. The control group received the same volume of citrate buffer. After 72 h of STZ administration, blood glucose concentration was determined (Accu-Chek sensor comfort, Roche Diagnostics). Ninety percent of rats reached a fasting serum glucose level over 250 mg/dL and were considered diabetic for further studies (*n* = 14). All rats were maintained for 30 days under a regular laboratory diet with water ad libitum (Figure 1 experimental design). After 30 days of follow-up, the treatments were started. The first group of diabetic animals with blood glucose values at 320 ± 16 mg/dL was administered only with vehicle solution and considered as diabetic control or untreated (DM group). The blood glucose values in the second group of diabetes (DA) were 355 ± 10 mg/dL, this group was treated with allicin (16 mg/kg/day by gavage). The treatments were administered daily by a month. At the end of the experiment (two months) and after completion of the experimental protocols, blood samples were collected. The animals were euthanized and skeletal muscle (Soleous) and the kidneys were surgically excised. Kidneys were dissected in cortex and medulla. All tissue samples were quickly frozen in liquid nitrogen and stored at −70 °C until further analysis.

### 2.3. Ethics Statement

This study was performed in accordance with the Guide for the Care and Use of Laboratory Animals, published by the U.S. National Institutes of Health, and approved by the Research Committee of the Instituto Nacional de Cardiología Ignacio Chávez (INC/CICUAL/001/2020) and by the Mexican Federal Regulation for animal experimentation and care (NOM-062-ZOO-1999) and for the disposal of biological residues (NOM-087-ECOL-1995).

### 2.4. Allicin Synthesis

Allicin was produced by oxidation of diallyl disulfide as previously reported [28]. For stabilization and storage, allicin was resuspended in water at 2.5% (*w/v*) and kept at −70 °C until it was used.

### 2.5. Blood Samples

Blood samples were obtained following an overnight fasting, and were collected on ice at 4 °C. At each time point (30 and 60 days), 1.0 milliliter of blood was taken from the caudal vein in conscious rats and collected in heparinized tubes. Blood samples were centrifuged at 1000× *g* for 15 min at 4 °C. The upper plasma phase was carefully pipetted and transferred into 1.5 mL microcentrifuge tubes and stored at −70 °C and used for the analysis of the total antioxidant status (TAS) and measurement of insulin levels.

### 2.6. Systolic Blood Pressure Record (SBP)

After 30 and 60 days of follow-up, the SBP was recorded in conscious, restrained rats by a validated tail-cuff plethysmography method (Narco Biosystems, Austin, TX, USA).

### 2.7. Plasma Biochemistry

The levels of blood urea nitrogen (BUN) and creatinine were measured in plasma samples using commercial kits according to the manufacturer’s instructions

### 2.8. Renal Function

To assess renal function, the rats were placed in metabolic cages (Nalgene, Rochester, NY, USA) to collect 24 h urine. Urine samples were centrifuged at 5000× *g* for 15 min to remove debris, and the supernatant was analyzed. The variables measured were diuresis, urea, creatinine, and the creatinine clearance.

### 2.9. Evaluation of α-SMA, CTGF, Epo, Epo-R, HIF-1α, Keap1, Nephrin, KIM-1, Nrf2, TGF-β1, and VEGF Expression in Renal Cortex

The kidney cortex was washed out thoroughly with ice-cold saline solution 10% *w/v* then homogenized in ice-cold 50 mM phosphate buffer pH 7.4 containing a cocktail of mammalian protease inhibitors using a Potter Elvehjem homogenizer. Protein concentration in homogenates was determined by the Bradford method using bovine serum albumin as a standard. Equal protein amounts (7.5 µg) were denatured in a gel loading buffer at 85 °C for 5 min, then loaded onto 10% SDS-polyacrylamide gels (SDS-PAGE), transferred to polyvinylidene difluoride (PVDF) membranes and incubated at 4 °C overnight with primary antibody diluted in Tris-buffered saline-Tween 0.1% solution. The protein bands were visualized with enhanced chemiluminescence reagents (Clarity Western ECL Substrate, Bio-Rad, Hercules, CA, USA) and their intensity was quantified using ImageJ (National Institutes of Health, Bethesda, MD, USA). Positive immunoreactive bands were quantified and expressed as the ratio of the protein of interest test sample to loading control in arbitrary units (a.u).

To assess urinary excretion of nephrin and KIM-1, sample volumes corresponding to 15 μg of total protein were precipitated on ice for 30 min with 10% (*w/v*) trichloroacetic acid in PBS. Samples were then centrifuged at 13,100× *g* for 10 min at 4 °C before washing pellets twice with ice-cold acetone. The samples were air-dried and dissolved in Laemmli buffer (62.5 mmol/L Tris–HCl (pH 6.8), 10% (*v/v*) glycerol, 2% (*w/v*) SDS, 5% (*w/v*) 2-mercaptoethanol and 0.05% (*w/v*) bromophenol blue) followed by heating at 95 °C for 5 min. Samples were loaded in SDS-PAGE gels as previously described.

### 2.10. Histological Assessment of Renal Injury

The kidneys from experimental groups were fixed in 10% formalin in PBS and embedded in paraffin. Sections (3 µm thick) were obtained and stained with periodic acid-Schiff (PAS) and Sirius Red. To evaluate the glomerular and mesangial areas, photomicrographs of PAS-stained sections were taken at a 200× magnification from non-overlapping fields. Thirty glomeruli were assessed in each rat kidney. Glomerular size and mesangial areas were determined by manually trace of the tuft perimeter and automatically measuring the PAS-positive material in each glomerulus with computerized image analysis software Axiovision Rel v. 4.8.2 (Carl Zeiss Microscopy, LLC., White Plains, NY, USA). The mesangial matrix index represented the ratio of mesangial matrix area divided by the tuft area. For interstitial fibrosis assessment, images of 10 cortex fields (200× magnification) were randomly recorded from Sirius Red-stained sections of each kidney. Interstitial fibrosis consisted in red stained areas (collagen I and III) [29] located between tubules and its quantification was performed using the same software mentioned above. Proportion of fibrosis was calculated by dividing the area of interstitial fibrosis by the total area per field, excluding the glomerular and luminal tubular areas. Microscopic images were obtained using a digital camera mounted on a light microscope (Axiophot2 Zeiss, Oberkochen, Germany). All slides were analyzed in a blinded fashion.

### 2.11. Evaluation of Oxidative Stress

To assess this parameter, we quantified the total antioxidant status (TAS) in plasma samples using a spectrophotometric method; also, we evaluated the expression of nuclear factor erythroid 2–related factor 2 (Nrf2) and kelch-like ECH associated-protein 1 (Keap1) in renal cortex by immunoblot assays. Further, we analyzed the expression of the hypoxia inducible factor 1α (HIF-1α), vascular endothelial growth factor (VEGF), erythropoietin (Epo), and erythropoietin receptor (Epo-R) as response proteins to hypoxic stimuli induced by oxidative stress.

#### Measurement of the Total Antioxidant Status (TAS)

TAS was determined using the 2,2′-Azino-bis(3-ethylbenzothiazoline-6-sulfonic acid) diammonium salt (ABTS) method. In brief, diluted plasma samples (5 μL) were mixed with a cationic ABTS solution (250 μL) prepared in phosphate buffer (50 mM pH 7.4) and the absorbance was recorded at 734 nm after 5 min using a Synergy HT multi-mode microplate reader (BioTek Instruments, Inc., Winooski, VT, USA). The results of TAS were expressed as Trolox equivalents (mmol/L).

### 2.12. Plasma Insulin Levels

To achieve this measurement, we used a commercially available Enzyme-linked Immunosorbent Assay kit, CEA448Ra, following the instructions of the supplier (USCN Life sciences Inc., Wuhan, Hubei, China).

### 2.13. Expression of GLUT4, IRS-1 and IRS-2 in Skeletal Muscle

Approximately 50 mg of wet muscle tissue was used to measure total crude membrane GLUT4 protein by immunoblotting. Muscle samples were homogenized in ice-cold RIPA lysis buffer (50 mM Tris-HCl, pH 8.0, 0.1% sodium dodecyl sulfate, 150 mM sodium chloride, 0.5% sodium deoxycholate and 1% NP-40). The supernatant was separated from debris by centrifugation at 16,000× *g* for 15 min at 4 °C. Fifteen micrograms of protein from each sample were separated using SDS-PAGE on a 12% acrylamide gel and transferred to a nitrocellulose membrane and later processed as described previously using monoclonal anti-GLUT4, IRS-1 and IRS-2 antibodies. Positive immunoreactive bands were quantified and expressed as the ratio of the protein of interest sample to loading control in arbitrary units.

### 2.14. Statistical Analysis

All data are expressed as means ± standard error of the mean (SEM). Comparisons amongst groups were made using one-way analysis of variance followed by Tukey’s test by using GraphPad version 5.00 (GraphPad Software, La Jolla, CA, USA). Differences were considered significant when *p* < 0.05.

## 3. Results

After 30 days of follow-up the diabetic rats showed hyperglycemia and lower body weight compared with Ctrl group (data not shown). To study the antioxidant effect of allicin on DN progression, the treatment with allicin was administered from day 30 to day 60.

### 3.1. Allicin Delays the Progression of Diabetic Nephropathy

Apart from albuminuria and GFR, established or potential risk markers for diabetic nephropathy include hyperglycemia, hypertension, tubular injury markers, presence of specific urinary peptides, and serum cystatin C levels. Thus, we assessed biochemical markers creatinine and blood urea nitrogen (BUN) in serum and creatinine and urea in urine. Further, the blood pressure and the expression of proteins involved in glomerular filtration barrier (nephrin), tubular injury (KIM-1), and fibrosis, (connective tissue growth factor (CTGF), transforming growth factor-β1 (TGF-β1), and alpha-smooth muscle actin (α-SMA)) were assessed as markers of progression of diabetic nephropathy.

At the end of the study, the groups with diabetes showed an increase in BUN. In addition, the urinary excretion of creatinine, urea, urinary volume and creatinine clearance, were higher in both diabetic groups than the control group. The allicin treatment partially but significantly decreased BUN, diuresis, and creatinine clearance (Table 1).

**Figure 2:**
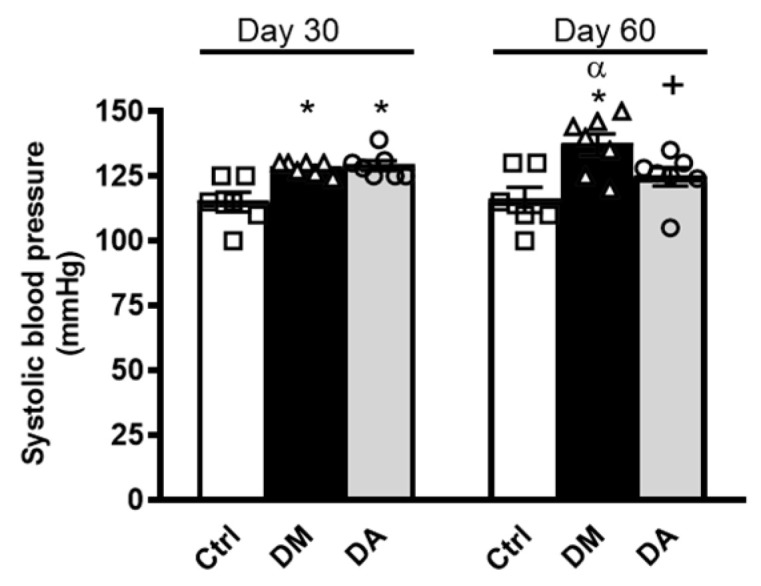
Effects of allicin on systolic blood pressure. Ctrl: control; DM: diabetic; DA: diabetic treated with allicin. mmHg; millimeters of mercury. Values represent mean ± SEM of at least 6 animals from each experimental group. * *p* < 0.05 vs. Ctrl; ^+^ *p* < 0.05 vs. DM; ^α^ *p* < 0.05 vs. DM day 30.

Before the treatment with allicin (day 30), SBP was increased in rats with diabetes compared with the control group (Figure 2). We did not find statistical differences between diabetic groups at day 30. At the end of the study (day 60), the SBP was significantly decreased in the DA group, whereas it was further increased in rats with DM without allicin treatment (Figure 2).

**Figure 3:**
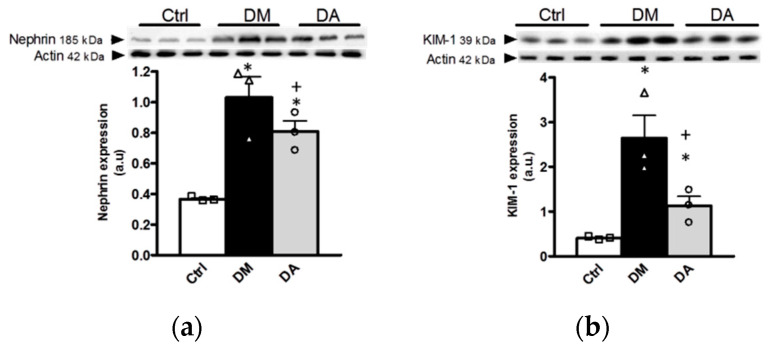
Effects of allicin on glomerular and tubular markers of kidney injury in cortex. (**a**) Nephrin and (**b**) kidney injury molecule-1 (KIM-1). Ctrl: control; DM: diabetes; DA: diabetes treated with allicin; a.u: arbitrary units. For representative Western blotting, 3 randomly selected samples per group were analyzed. Results are presented as mean ± SEM and analyzed by one-way ANOVA. Statistical significance was established as * *p* < 0.05 vs. Ctrl, ^+^ *p* < 0.05 vs. DM.

In the renal cortex the protein expression of nephrin, and KIM-1 were used as biomarkers of progression of DN. The data highlighted a higher expression of nephrin and KIM-1 in kidneys from groups with diabetes when compared with control group (Figure 3a,b), which suggested renal injury at glomerular and tubular levels in animals with diabetes. Although the allicin treatment decreased significantly the expression of nephrin and KIM-1, the renoprotective effect was partial (Figure 3a,b).

In addition, we assessed the urinary excretion of glomerular (nephrin) and tubular (KIM-1) injury markers. We observed an important increase in the urinary excretion of nephrin and KIM-1 in the diabetic group (Figure S1) when compared with the control group. The allicin treatment attenuated the effect induced by diabetes (Figure S1).

### 3.2. Histopathological Analysis

We previously studied the histopathological alterations in the kidney of the experimental model of diabetes used in the present study [12]. The previous report showed that two of the main structural alterations of DN, such as mesangial expansion and interstitial fibrosis, are presented in the animal model with one month of diabetes [12]. Considering this background, in the present report we analyzed the possible effect of allicin as a potential therapeutic option to reverse, delay or prevent the progression of DN.

**Figure 4:**
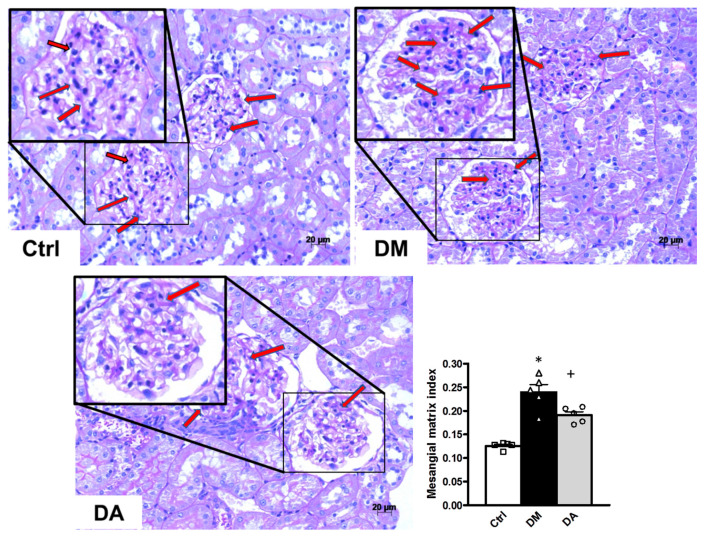
Mesangial matrix and quantitative analysis of rat kidneys. Representative photomicrographs of one kidney cortex section of each experimental group. Control (Ctrl) with no changes in glomeruli mesangium seen in light purple (arrows). Diabetes (DM) illustrates expansion of the mesangium (arrows), which is thicker and denser than the one of control group. Diabetes + Allicin (DA) depicts a mesangium (arrows) with characteristics between the control and the DM groups. Periodic acid-Schiff (PAS stain), 200× original magnification. Graph shows quantitative analysis of mesangial matrix index. Values are expressed as mean ± SEM of *n* = 5 * *p* < 0.001 vs. Ctrl; ^+^ *p* < 0.001 vs. DM.

**Figure 5:**
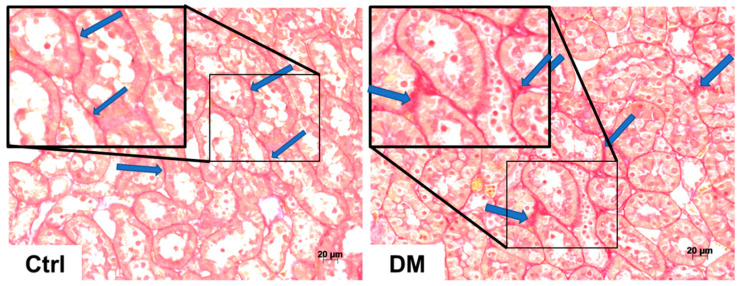

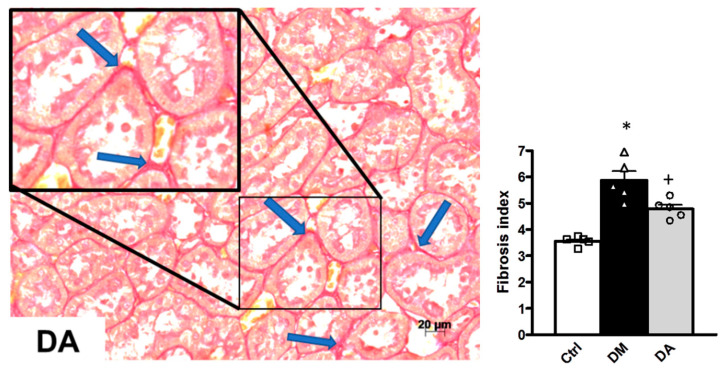
Interstitial fibrosis and quantitative analysis of rat kidneys. Sirius Red for collagen staining (fibrosis) in red of kidney cortex sections, representative of each experimental group. Control (Ctrl) shows very thin red lines (arrows) separating the cortex tubules in a normal interstitium. Diabetes (DM) depicts a clear modest widening of the interstitium (arrows) in comparison with the control. Diabetes + Allicin (DA) photomicrograph allows the visualization of a near normal interstitium, with a few areas of a thicker space between tubules (arrows). Original magnification 200×. Graph shows quantitative analysis of interstitial fibrosis. Values are expressed as mean ± SEM of *n* = 5 * *p* < 0.001 vs. Ctrl; ^+^ *p* < 0.001 vs. DM.

Our morphometric study demonstrated that the glomerular area was not significantly different in the three experimental groups (data not shown), but the mesangial matrix index was significantly elevated in the groups with diabetes (DM and DA groups) in comparison to that of the control group (* *p* < 0.001) (Figure 4). The increase in the mesangial matrix index was suppressed in the DA group compared with the DM group (^+^ *p* < 0.001) (Figure 4). On the other hand, the tubular interstitial fibrosis assessment showed that diabetes induced an increase in this parameter. The interstitial fibrosis index was significantly increased in the renal cortex of D group (Figure 5). We also observed less interstitial fibrosis in the group with diabetes treated with allicin (^+^ *p* < 0.001) (Figure 5).

**Figure 6:**
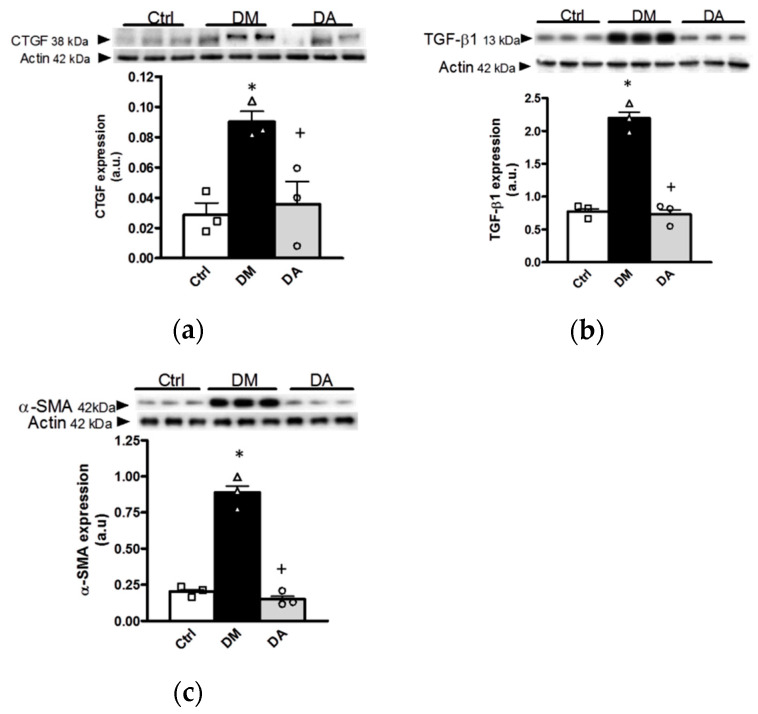
Effects of allicin on fibrotic markers in kidney. (**a**) Connective tissue growth factor (CTGF), (**b**) transforming growth factor-β1 (TGF-β1) and (**c**) alpha-smooth muscle actin (α-SMA). Ctrl: control; DM: diabetes; DA: diabetes treated with allicin; a.u: arbitrary units. For representative Western blotting, 3 randomly selected samples per group were analyzed. Results are presented as mean ± SEM and analyzed by one-way ANOVA. Statistical significance was established as * *p* < 0.05 vs. Ctrl, ^+^ *p* < 0.05 vs. DM.

To support the histological analyses, in the renal cortex homogenates from the experimental groups were evaluated the expression of the pro-fibrotic markers, connective tissue growth factor (CTGF), transforming growth factor-β1 (TGF-β1), and alpha-smooth muscle actin (α-SMA). In the kidney from rats with diabetes, the results showed an increase in the expression of the three fibrotic markers (Figure 6a–c)—Allicin treatment prevented such alterations (Figure 6a–c).

### 3.3. Mechanisms Associated With the Beneficial Effect Exerted by Allicin in Diabetic Nephropathy

#### 3.3.1. Effects of Allicin on Oxidative Stress

The antioxidant effects of allicin were assessed by measurements of TAS in plasma and expression of Nrf2/Keap1 in the renal cortex. In addition, we analyzed the expression of response proteins sensitive to hypoxia, induced by the oxidative stress in this outer portion of kidney.

**Figure 7:**
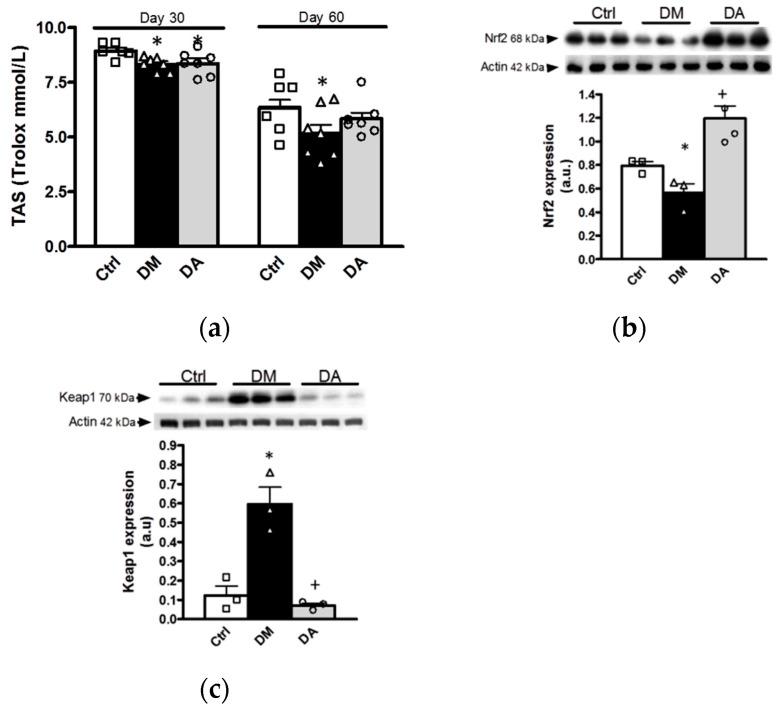
Evaluation of oxidative stress. (**a**) Total antioxidant status in plasma, (**b**) nuclear factor erythroid 2-related factor 2 (Nrf2) expression in kidney cortex, and (**c**) kelch-like ECH associated-protein 1 (Keap1) expression in kidney cortex. Ctrl: control; DM: diabetes; DA: diabetes treated with allicin; a.u: arbitrary units. For representative Western blotting, 3 randomly selected samples per group were analyzed. Results are presented as mean ± SEM and analyzed by one-way ANOVA. Statistical significance was established as * *p* < 0.05 vs. Ctrl, ^+^ *p* < 0.05 vs. DM.

Before starting allicin treatment, plasma TAS of diabetic rats (DM and DA groups) was numerically decreased at 30 days when compared with control rats; however, we did not find statistical differences between groups at this time. After 60 days of follow up, the TAS further decreased in the plasma of the group with untreated diabetes when compared with the control group (Figure 7a). Allicin treatment showed a trend to prevent the effect of diabetes although without reaching statistical significance (Figure 7a).

The data obtained at 60 days of follow-up were associated to an impaired ratio of Nrf2/Keap1; thus, Nrf2 expression was decreased in kidney cortex obtained from diabetic group when compared to control group (Figure 7b). In contrast, the expression of Keap1 was increased in the kidney cortex of the diabetic group (Figure 7c).

Allicin treatment prevented the effects induced by diabetes. Thus, Nrf2 protein expression was increased, while Keap1 was decreased in the renal cortex of DA rats when compared with of the diabetic rats. In summary, allicin improved plasma TAS and reversed the changes in Nrf2 and Keap1 expression, showing its renal antioxidant effects on diabetes.

#### 3.3.2. Effect of Allicin on Markers of Hypoxia in Renal Cortex

OS through modulation of other pathophysiological pathways is one of multiple molecular mechanisms involved in the progression of DN. It has been hypothesized that OS modulates hypoxic factors that start mechanisms associated to the progression of renal diseases. Therefore, to unravel further effects of allicin on OS and its associated pathophysiological pathways, we assessed the expression of HIF-1α, VEGF, Epo and Epo-R, a group of proteins closely related with the hypoxic response induced by oxidative stress.

**Figure 8:**
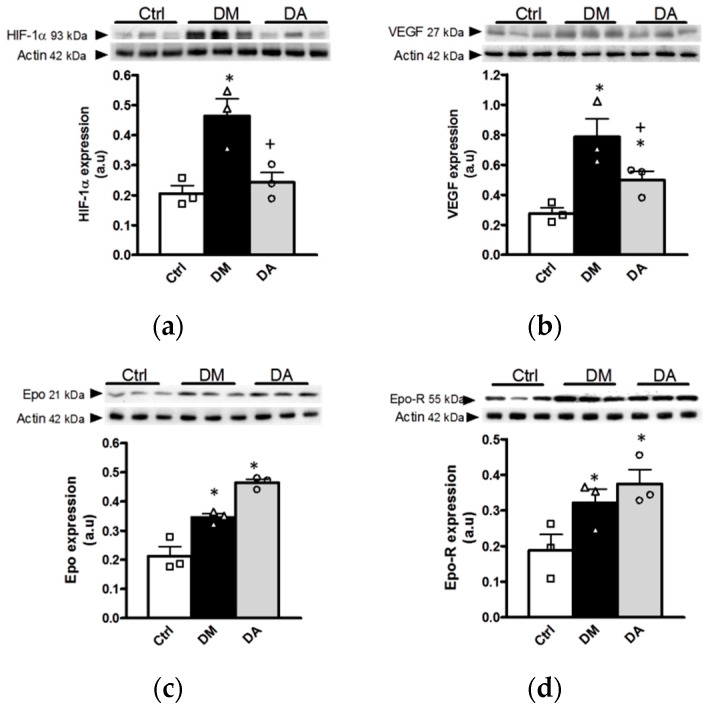
Effects of allicin on hypoxic factors induced by oxidative stress. (**a**) Hypoxia-inducible factor 1-alpha (HIF-1α), (**b**) vascular endothelial growth factor (VEGF), (**c**) erythropoietin (Epo), and (**d**) erythropoietin receptor (Epo-R). Ctrl: control; DM: diabetes; DA: diabetes treated with allicin; a.u: arbitrary units. For representative Western blotting, 3 randomly selected samples per group were analyzed. Results are presented as mean ± SEM and analyzed by one-way ANOVA. Statistical significance was established as * *p* < 0.05 vs. Ctrl, ^+^ *p* < 0.05 vs. DM.

The expression of HIF-1α, a key factor involved in the hypoxia response, was increased in renal cortex from in the diabetic group (Figure 8a). In addition, diabetes also significantly increased the VEGF, Epo and Epo-R compared to control group (Figure 8b,c). Although the allicin treatment prevented the increase in HIF-1α and VEGF in comparison with the diabetic group (Figure 8a,b), this treatment further increased Epo and Epo-R without statistical difference between both groups of diabetes (Figure 8c,d).

### 3.4. Effects of Allicin on Glucose Homeostasis

Diabetes is characterized by increased appetite (polyphagia) concomitant to decreased uptake and utilization of glucose by peripheral tissues; such effects are due to the lack of insulin or because the insulin pathway signaling is impaired. All these metabolic abnormalities are responsible for the maintenance of hyperglycemia as well as the loss of body weight and the development of long lasting microvascular and macrovascular complications. The reported beneficial effects in progression of DN observed in the present studies were likely induced by the antioxidant properties of allicin. However, we do not rule out the participation of other beneficial mechanisms exerted by allicin during diabetes. Thus, to address other probable beneficial effects of allicin, and to better understand this issue, we quantified glucose and insulin concentrations in plasma and as well as the expression of GLUT4, IRS-1 and IRS-2 in skeletal muscle from our experimental groups.

**Figure 9:**
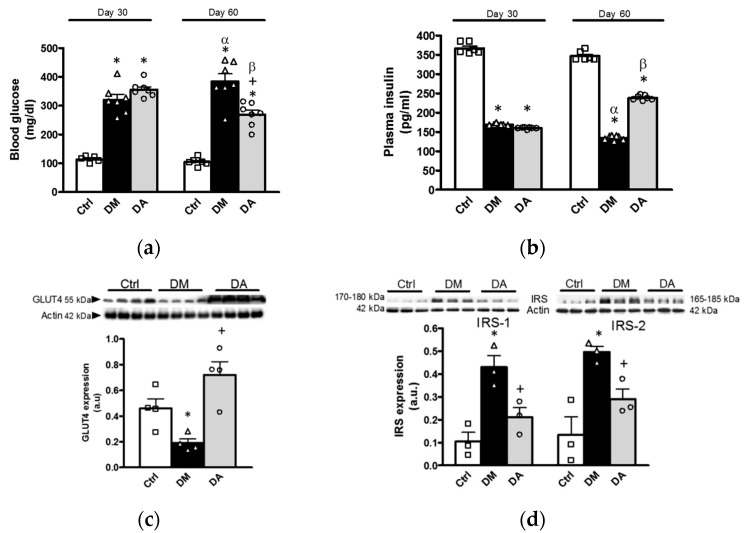
Effects of allicin on glucose homeostasis of rats. (**a**) Blood glucose and (**b**) plasma insulin levels; (**c**) glucose transporter expression and (**d**) insulin receptor substrate expression were assessed in skeletal muscle homogenate. Ctrl: control; DM: diabetic; DA: diabetic treated with allicin; a.u: arbitrary units. For representative Western blotting, 3 randomly selected samples per group were analyzed. Results are presented as mean ± SEM and analyzed by one-way ANOVA. Statistical significance was established as * *p* < 0.05 vs. Ctrl, ^+^ *p* < 0.05 vs. DM, ^α^ *p* < 0.05 vs. DM day 30, ^β^ *p* < 0.05 vs. DA day 30.

Before the treatment with allicin (day 30), blood glucose concentrations in rats with diabetes were significantly higher than the control group (Figure 9a). We did not find statistical differences between diabetic groups. These data were associated with the lower concentration of insulin in the two experimental groups with diabetes compared with control group (Figure 9b). At the end of the study (day 60), the blood glucose concentration in the group with diabetes was further increased (Figure 9a). In contrast, the blood glucose concentration in DA group was decreased in comparison to diabetic group and with the glucose values before starting the treatment (Figure 9a). Likewise, in diabetic group plasma insulin was lower compared with control group and with the insulin levels before starting the treatment with allicin (Figure 9b). DA group showed increase in insulin levels compared with the group of diabetes.

These data from hyperglycemia and decreased insulin levels in diabetes were concordant with the low expression of GLUT4 observed in muscle of diabetic animals (Figure 9c). The expression of IRS-1 and IRS-2 were also increased in muscle from diabetes group when compared with control group (Figure 9d). Interestingly, the expression of GLUT4 was significantly increased upon the treatment with allicin and the increases in IRS-1, and IRS-2 expression were prevented when diabetic rats were treated with allicin (Figure 9d).

## 4. Discussion

In patients with diabetes, the diagnosis and treatment of kidney disease are made when several years of diabetes have elapsed and usually when the disease is advanced. In order to translate a clinical situation to basic research, we used a 30-day diabetic experimental model that induces structural renal damage as we have previously demonstrated [12]. Thus, the data obtained from our research is from the therapeutic role perspective instead of a preventive standpoint, as it has been shown in other works [24,26]. The present study aimed to assess if the antioxidant garlic-derived compound allicin can reverse or delay the progression of established DN. We found that allicin effectively arrested the progression of nephropathy throughout several mechanisms including antihypertensive, antioxidant, antifibrotic, and antidiabetic pathways. Besides, allicin shows to modulate hypoxia response proteins that are likely associated with OS and play a key role on fibrosis during diabetes.

Current treatments to manage diabetes, hypertension, and oxidative stress as key risk factors for CVD and chronic complications are partially successful and there is still a large residual risk to develop vascular complications and diabetic kidney disease. Therefore, the novel agents targeting pathophysiological mechanisms such as hyperglycemia, hypertension, OS, hyperfiltration, inflammation or fibrosis, have been a major focus for the development of new treatments. In this context, a major factor responsible for the development of chronic complications during diabetes is the increase in OS [1,30]. Evidence from clinical and experimental studies has showed that increase in OS plays a key role in the initiation and progression of diabetic complications [9,10,11,12,13,14]. Hyperglycemia during diabetes contributes to OS through free radicals generation and suppression of the antioxidant defense systems which exacerbate OS.

In the progression of diabetic nephropathy, patients have a higher prevalence of risk factors for CVD including hypertension, hyperglycemia, inflammation, and oxidative stress. Even, when patients have hypertension and diabetes, which is a common combination, their risk of developing CVD doubles [1]. Additionally, it has been described that chronic inflammation in association with increased formation of reactive oxygen species (ROS) and alteration of nitric oxide signaling is a key mechanism that links kidney and CVD, particularly in the context of diabetes [30]. Besides, a meta-analysis previously reported that the benefit of lowering blood pressure levels includes reduction of the risk of CVD, coronary heart disease, stroke, diabetes, heart failure, and the progression of albuminuria [31]. This suggests that the control of the risk factors present during diabetes could reduce the risk of mortality due to vascular complications.

The progression of kidney disease is gradual and the changes in thickening of the glomerular basement membrane, capillary and tubular basement membrane thickening, loss of endothelial fenestrations, mesangial matrix expansion, and loss of podocytes with effacement of foot processes are the main histological alterations in kidney structure [13]. In this context the increase in the expression of profibrotic proteins including CTGF, α-SMA, TGF-1β, fibronectin, or collagen plays a preponderant role [32,33,34]. In patients with advanced type 2 DN, the expression of CTGF was found not only in the glomerular cells, vascular endothelial cells, and interstitial cells, but also in the epithelial cells of various tubular segments in the diabetic kidney and correlated with proteinuria, serum creatinine, and interstitial fibrosis [35]. Similarly, our study showed alterations in biochemical and urinary markers of renal function, increase in SBP, mesangial matrix index and interstitial fibrosis that were associated to an increase in the protein expression of injury and fibrotic markers in kidneys of the group with diabetes. Our results indicate that the allicin treatment could delay diabetic nephropathy through its effects on blood pressure levels, podocyte and tubule injury, through modulation of nephrin and KIM-1 expression. These results were concordant with the attenuation of mesangial matrix index, the interstitial fibrosis and with the expression of CTGF, α-SMA, and TGF-β1.

In our study, we observed an increase in the expression of nephrin in the renal cortex, which was associated with its increased urinary excretion. Other studies in hypertensive patients and experimental models have described increased nephrin expression [36,37,38,39] Even in patients with CKD, loss of nephrin occurs despite the increased level of nephrin mRNA [36]. In contrast, other studies report that the expression of nephrin is reduced [36,37]. Studies in patients showed that the urinary excretion of nephrin can be a good marker of kidney damage since nephrinuria occurs even without albuminuria [40,41]. We hypothesize that the increase in the nephrin expression may represent a compensatory mechanism that allows restoring the nephrin loss through the urine, as our results showed. However, this protective mechanism of the glomerular filtration barrier cannot be maintained in the long term and will eventually be inefficient, thus, the expression of nephrin will gradually decrease, and the integrity of the glomerular filtration barrier will be lost. A weakness of our study is that we did not evaluate other integral proteins of the filtration barrier, but we did not deny that their expression was impaired.

Previously, we demonstrated that allicin administered during the development of CKD conferred nephroprotection through the modulation of antihypertensive and antioxidant mechanisms [23,24]. It is well known that alterations in Nrf2/Keap1 pathway are associated to OS and decreased endogenous antioxidants enzymes such as catalase, glutathione peroxidase, heme oxygenase, and superoxide dismutase [12,24]. Here, we demonstrated that allicin is able to improve the TAS as well as Nrf2/Keap1 ratio in diabetes. This findings can be supported by previous reports describing that allicin increased the levels of intracellular glutathione by the up-regulation of phase II detoxifying enzymes in a Nrf2/Keap1 dependent pathway (hemeoxygenase-1, superoxide dismutase, glutathione peroxidase, glutathione-S-transferases, NAD(P)H-quinine oxidoreductase, γ-glutamylcysteine synthetase) [23,42,43]. Moreover, the antioxidant mechanisms of allicin include direct antioxidant effects by preventing the formation of free radicals [44,45,46] and lipid peroxidation through hydroxyl and peroxyl radicals scavenging by transferring its allylic hydrogen to the oxidized substrate [47,48]. Allicin reacts rapidly with free thiol groups in molecules such as glutathione and cysteine residues in proteins [49], hence oxidation of protein thiols can lead to protein structure changes that result in loss or gain of function [22].

**Figure 10:**
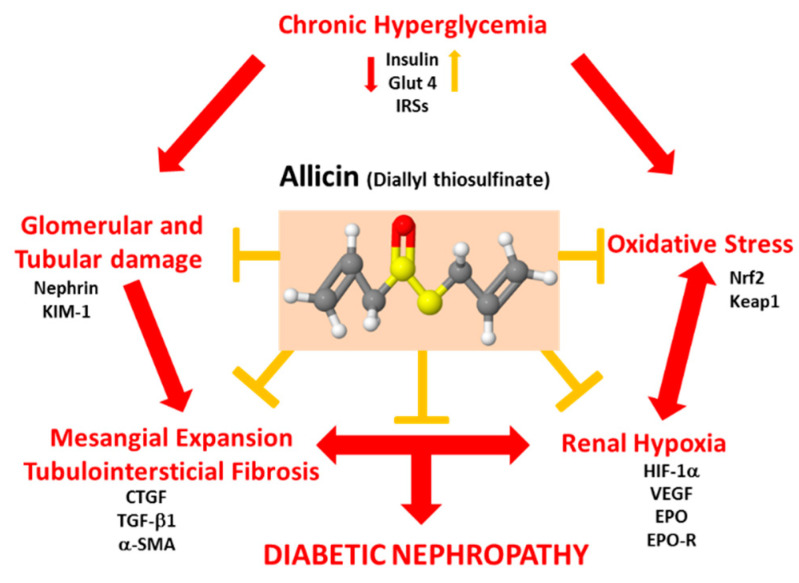
Effects of allicin on pathophysiological mechanisms during progression of nephropathy associated to diabetes. Structure of allicin (diallyl thiosulfinate): red spheres, oxygen; yellow spheres, sulfur; gray spheres, carbon; white spheres, hydrogen.

Hyperglycemia does not only promote OS, but also results in decreased intrarenal oxygenation [15,50]. It is known that reduced oxygen tension can promote the expression of hypoxia inducible factors (HIFs) to counteract hypoxia, preventing ROS overproduction (Figure 10). However, HIF-1α may also exert adverse responses, such as the aggravation of diabetic retinopathy through the induction of VEGF [51]. Our results show an increase in HIF-1α expression in the diabetic group that was associated with the expression of hypoxia-inducible proteins such as VEGF, Epo and its receptor Epo-R. Allicin decreased HIF-1α and VEGF, while in contrast, it increased Epo and Epo-R in renal cortex. It is well known that Epo has renoprotective, antioxidant and antiapoptotic effects, that protect peritubular capillaries and prevent glomeruloesclerosis [52,53,54]. In addition, Epo and the activation of its receptor decrease albuminuria, mesangial expansion and renal fibrosis [55,56,57]. Other studies have shown that overexpression of HIF-1α in tubular epithelial cells contributes to the progression of renal fibrosis, and the inhibition of HIF-1α expression prevents the progression of renal fibrosis and attenuated DN progression [19,58,59].

Our data suggest that OS-induced hypoxic response may play an important role in the overexpression of tubular and glomerular markers of damage (nephrin, KIM-1) as well as in the renal structural damage (mesangial matrix and interstitial fibrosis) induced by diabetes. In this regard, it has been reported that allicin prevents myocardial fibrosis by blocking CTGF and TGF-β1 protein expression during diabetes in a dose-dependent manner. Furthermore allicin reduced blood glucose levels and improved cardiac function [60]. In vitro, allicin inhibits the α-smooth muscle actin (α-SMA), vimentin and collagen type 1 expressions, as well as the ERK1/2-TGF-β1 signaling pathway [34]. It is well known that this signaling pathway plays a key role in epithelial–mesenchymal transition (EMT) and tubulointestitial fibrosis in DN [34]. In another study, the administration of allicin immediately after hyperglycemia confirmation prevented renal hypertrophy, ultrastructural damage of the glomerular endothelium, and thickening of the basement membrane. The authors suggested that the nephroprotective effect of allicin was dependent on the TGF-β1/ERK 1/2 pathway [26]. Likewise, it is known that Nrf2 is capable of conferring antifibrotic effects mediated by the inactivation of PI3K/Akt signaling pathway [61]. Therefore, we did not discard that allicin, through modulation of Nrf2-associated signaling pathways, decreases mesangial expansion and fibrosis (Figure 10).

Diabetes is characterized by hyperglycemia caused by defects in secretion and/or insulin signaling, which is directly associated with alterations in the glucose uptake and signaling of insulin-dependent glucose transporters (GLUT) (Figure 10). The model of streptozotocin-induced diabetes produces the partial destruction of beta cells in pancreas, therefore significantly reducing the synthesis and secretion of insulin into the circulation. Our results showed a reduction in blood glucose levels in allicin-treated diabetic group when compared with untreated diabetic group, suggesting that allicin may improve the handling of glucose by insulin-dependent organs. Thus, we assessed the mechanism responsible for the glucose uptake in skeletal muscle, an insulin-dependent tissue. In diabetic rats, the GLUT4 protein expression was decreased; in contrast, the insulin receptor substrate 1 (IRS-1) and IRS-2 protein expressions were increased in spite of the partial loss of insulin levels. It has been described that IRS-2 is suppressed at the transcriptional level by insulin; therefore, the mRNA of IRS-2 increases in the fasting state, when the serum insulin levels are quite low, and immediately decreases after food intake, associated with the increase in the serum insulin levels induced by food intake [62]. In contrast, hyperinsulinemic state downregulates IRS protein expression such as occurs in metabolic syndrome [62]. Our experimental diabetic model showed low levels of insulin that could explain hyperglycemia, a decrease in GLUT4 protein expression (Figure 9) and an increase in IRSs protein expression. Allicin was able to partially prevent the alterations in insulin signaling, which was traduced in reduction in hyperglycemia. Moreover, allicin treatment also improved insulin concentrations, suggesting that this compound might have beneficial effects on the remnant pancreatic beta cells. Other beneficial effects of allicin include the improvement in blood glucose, fructosamine, glycated hemoglobin, triglycerides, total cholesterol, high/low-density lipoprotein levels, and reduction in body weight gain and fat deposition [63,64]. On the other hand, it has been proposed that the imbalance in the intestinal microbiota may play a key role in the absorption of nutrients and therefore in the development of metabolic diseases. In this regard, it was shown that the effects of allicin on obesity-associated dyslipidemia are related to the modification of the intestinal microbiota [64].

Besides, oxidative stress, inflammation, and vascular remodeling actively participate in the progression of diabetic macroangiopathy, and allicin blocks these pathogenic processes by decreasing tumor necrosis factor α (TNF-α), vascular cell adhesion molecule 1 (VCAM-1), matrix metalloproteinase 2 (MMP-2), inducible nitric oxide synthase (iNOS), and monocyte chemotactic protein 1 (MCP-1), possibly in association with its effects on nuclear factor kappa-B (NF-κB), Nrf2 and blood glucose [33].

Alliin, a sulfur compound precursor and natural precursor of allicin, has shown several properties that could improve glucose metabolism, such as inhibition of glycolysis or direct/indirect stimulation of insulin secretion; it has been suggested that its hypoglycemic activity can be due to this sulfur compound [63,65,66]. In this manner, in agreement with our results, we cannot rule out that allicin has antidiabetic activity because sulfur is also present in the allicin structure (Figure 10). In this regard, a recent analysis of physicochemical properties of allicin revealed that this compound displays good permeation and molecular docking studies predict that allicin has the potential to bind at the active site of sulfonylurea receptor 1 (SUR1), complex 1 and peroxisome proliferator-activated receptor-gamma (PPARγ) which could promote antidiabetic properties of allicin [67]. Studies addressing the expression and activity of these receptors under allicin treatment will shed experimental light on this issue.

Thus, our results suggest an antidiabetogenic mechanism of allicin which involves partially an insulin secretagogue mechanism and a signaling pathway that modulates GLUT4 and IRS, a complex signaling pathway that was not addressed in our study and will be studied in future research. In addition, our lysis methods in muscle do not discriminate between cytosol and membrane fractions. Therefore, our results show the increase in GLUT4 expression, but this does not indicate its expression at the plasma membrane or cytoplasmic, nor does it suggest IRSs activation or translocation. Another limitation of our study is that we do not know how the expression of GLUT4 and the IRSs were previous to administration of allicin. These data at the same time strengthen the present study because our experimental design allows us to claim that the effects found in the treated group were induced by allicin. Our study was designed to simulate what happens in patients who are usually diagnosed and treated when the disease is already in course and several risk factors for vascular disease are already established [1,12,30]. Hence, our results support noticeable antihypertensive and antidiabetic effects of allicin, which will be deeply addressed in future work. The beneficial effects of allicin have also been described in degenerative diseases such as cancer. Thus, allicin has a potent inhibitory activity on ornithine decarboxylase, a rate-limiting enzyme involved in oncometabolite biosynthesis. In addition, in assays in vitro, allicin has been shown to inhibit cell proliferation and induce apoptosis [68].

In addition, our data showed novel effects of allicin in diabetes suggesting that this compound could be potentially considered as a new therapeutic option targeting the OS–hypoxia–fibrosis pathway in diabetes (Figure 10). We need to conduct a research experiment to obtain the information about the allicin activity as antidiabetic agent, especially related to the quantity of this compound with intrinsic antidiabetic activity.

Diabetes, hypertension, hyperglycemia and oxidative stress are crucial risk factors for CVD and diabetic kidney disease and often coexist. Thus, the patients require therapy for each of these alterations in order to reduce or prevent the vascular complications. Still, treatments can help to relieve the symptoms and slow the progression, thereby offering a better prognosis and life quality to patients. However, these interventions do not prevent the progression of vascular diseases. Probably, the reason for this is that there are various etiologies and mechanisms involved, hence multi-target interventions should be used. Our results showed that allicin can offer several beneficial effects, such as antihypertensive, antioxidant, anti-inflammatory, antifibrotic, and antidiabetic, that globally contribute to delay the progression of nephropathy and probably the risk for CVD. Therefore, allicin may be considered as a potential natural therapeutic option to support the pharmacological treatment of vascular and renal diseases, not only secondary to diabetes, but also the vascular diseases induced by other causes such as hypertension and oxidative stress.

## 5. Conclusions

Based on the presented results, allicin delays the progression of nephropathy, likely mediated by the downregulation of the OS–hypoxia–fibrosis pathway, HIF-1α and CTGF. Furthermore, allicin improved blood pressure, insulin bioavailability and decreased hyperglycemia by the modulation of GLUT4 and IRS proteins in muscle. Thus, we can suggest allicin as a therapeutic natural option to aid in the improvement of hyperglycemia, control of OS and the prevention of DN progression.

## Figures and Tables

**Table 1:** Biochemical markers of renal function.

Parameter/Group	Ctrl	DM	DA
Urinary volume (mL/24 h)	16.44 ± 2.10	44.67 ± 6.97 *	33.18 ± 2.53 *^+^
sCr (mg/dL)	0.69 ± 0.01	0.73 ± 0.04	0.73 ± 0.01
BUN (mg/dL)	22.83 ± 1.108	30.44 ± 0.88 *	27.60 ± 1.22 *
uCr (mg/mL)	7.44 ± 0.69	28.88 ± 5.96 *	22.50 ± 2.72 *
uUrea (mg/mL)	160.6 ± 12.77	323.4 ± 21.36 *	256.5 ± 18.83 *^,+^
CrCl (ml/min)	0.75 ± 0.016	2.66 ± 0.20 *	2.15 ± 0.08 *^,+^

Ctrl: control; DM: diabetes mellitus; DA: diabetes allicin; sCr: serum creatinine; BUN: blood urea nitrogen; uCr: urinary creatinine; uUrea: urinary urea; CrCl: creatinine clearance. * *p* < 0.05 vs. Ctrl; ^+^ *p* < 0.05 vs. DM.

## Supplementary Materials

The following are available online at https://www.mdpi.com/2076-3921/9/11/1134/s1, Figure S1: Urinary excretion of (a) Nephrin and (b) KIM-1. Ctrl: Control; DM: Diabetes; DA: Diabetes treated with allicin. a.u.; arbitrary units. Values are expressed as mean ± SEM of 5 animals from each experimental group * *p* < 0.05 vs. Ctrl, + *p* < 0.05 vs. DM.
