# Supplementary material for: Effects of Allicin on Pathophysiological Mechanisms during the Progression of Nephropathy Associated to Diabetes
Source: Antioxidants (Basel). 2020 Nov 15;9(11):1134. doi: 10.3390/antiox9111134 (PMC7697950; doi:10.3390/antiox9111134)
Supplement: Supplementary file 1 [file antioxidants-09-01134-s001.pdf]

antioxidants

MDPI

Supplementary Figure

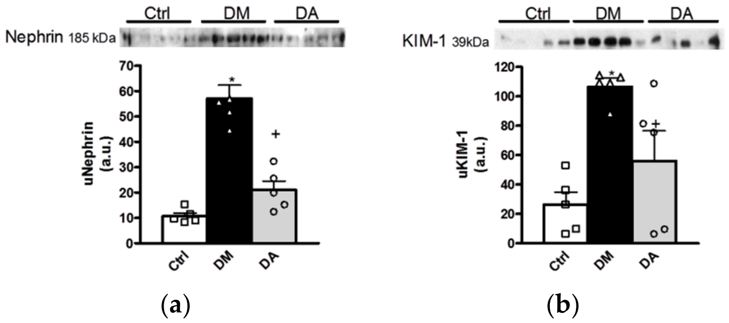

Figure S1. Urinary excretion of (a) Nephrin and (b) KIM-1. Ctrl: Control; DM: Diabetes; DA: Diabetes treated with allicin. a.u.; arbitrary units. Values are expressed as mean ± SEM of 5 animals from each experimental group * p <0.05 vs Ctrl, + p <0.05 vs DM.
